# *In Situ* Aquaculture Methods for *Dysidea avara* (Demospongiae, Porifera) in the Northwestern Mediterranean

**DOI:** 10.3390/md8061731

**Published:** 2010-05-26

**Authors:** Sonia de Caralt, Javier Sánchez-Fontenla, María J. Uriz, Rene H. Wijffels

**Affiliations:** 1 Centre d’Estudis Avançats de Blanes (CEAB-CSIC), Accés a la Cala St Francesc 14, Blanes 17300, Spain; E-Mails: jsmf77@gmail.com (J.S.-F.); iosune@ceab.csic.es (M.J.U.); 2 Bioprocess Engineering Group, Department of Agrotechnology and Food Science, Wageningen University, P.O. Box 8129, 6700 EV Wageningen, The Netherlands; E-Mail: Rene.Wijffels@wur.nl (R.H.W.)

**Keywords:** sponge culture, growth, survival, bioactivity, secondary metabolites

## Abstract

Marine sponges produce secondary metabolites that can be used as a natural source for the design of new drugs and cosmetics. There is, however, a supply problem with these natural substances for research and eventual commercialisation of the products. *In situ* sponge aquaculture is nowadays one of the most reliable methods to supply pharmaceutical companies with sufficient quantities of the target compound. In this study, we focus on the aquaculture of the sponge *Dysidea avara* (Schmidt, 1862), which produces avarol, a sterol with interesting pharmaceutical attributes. The soft consistency of this species makes the traditional culture method based on holding explants on ropes unsuitable. We have tested alternative culture methods for *D. avara* and optimized the underwater structures to hold the sponges to be used in aquaculture. Explants of this sponge were mounted on horizontal ropes, inside small cages or glued to substrates. Culture efficiency was evaluated by determination of sponge survival, growth rates, and bioactivity (as an indication of production of the target metabolite). While the cage method was the best method for explant survival, the glue method was the best one for explant growth and the rope method for bioactivity.

## 1. Introduction

Since the beginnings of the marine chemical ecology in the 1940s sponges turned out to be interesting organisms to study for commercial applications. Sponges contain secondary metabolites, which play important ecological roles in nature such as deterring fish from predation or inhibiting settlement and growth of foulants [1–3] and have been shown to have biomedical properties. Many secondary metabolites from sponges have been reported to inhibit cellular growth and are therefore interesting natural products for obtaining new drugs against cancer [4,5]. Lately, new pharmacological properties of sponge secondary metabolites have been discovered, such as their capacity to inhibit the nuclear transcription factor-κB (NF-κB), which is one of the principal inducible transcriptional factors that plays a critical role in cancer development and in inflammation (see Folmer [6], for a review on NF-κB inhibitors). This recent discovering makes sponges, even more interesting targets in the drug discovery field than previously thought.

The sponge *Dysidea avara* produces the sesquiterpene hydroquinone avarol and its corresponding quinone avarone. These secondary metabolites are cytostatic agents with potent anti-leukemic [7], anti-viral, and anti-inflammatory activities. Recently, the NF-κB inhibitor activity of avarol has been described [8] and might have an essential function in these observed anti-viral and anti-cancer activities. Moreover, avarol presents a moderate antibacterial activity against Gram-positive strains, and anti-fungal activities against a limited range of microorganisms [9]. Furthermore, it inhibits HIV-1 reverse transcriptase [10,11] and also is the main component (e.g., 60–98 wt.%) of a skin cream for treating psoriasis [12].

Unfortunately, enormous quantities of avarol, higher than what can be found in the natural sponge populations, are necessary for its pharmaceutical applications. To overcome this supply problem, different approaches have been assayed. The biomedical potential of *Dysidea avara* has generated a wide variety of studies about different culture techniques based on this sponge species. Attempts to establish cell cultures of *D. avara* have also been performed based on both cell suspensions and cell aggregates (primmorphs) [13–17]. Another original *ex situ* method, which showed high survival and growth rates, was to grow sponge juveniles from larvae [18]. Although the results obtained until now are encouraging, more investigation is required to make the *ex situ* culture methods a real possibility to produce the secondary metabolites in sufficient quantities to meet the market needs. On the other hand, the private enterprise KliniPharm GmbH is culturing *D. avara* in the Eastern Mediterranean Sea by holding explants on ropes to obtain avarol for the market (http://www.klinipharm.com). However, only few growth data of those farmed explants have been published [19]. Although at present the most reliable method to culture sponges is *in situ* aquaculture [20,21] more studies are required to optimize the whole process.

Sponge aquaculture was originally based on the practice to culture bath sponges (*i.e.*, genera *Coscinoderma*, *Hippospongia* and *Spongia*) by holding sponge cuttings on ropes [22–25]. Bath sponges have a keratose skeleton made of a network of spongine fibres providing them with a consistent structure [21]. When culturing other sponge species that have less structural elements with a less resistant skeleton it is more appropriate to use meshes instead of ropes [26–28].

In this study three different *in situ* experimental culture methods for growing *Dysidea avara* (Schmidt, 1862) have been tested in order to select the best one to be applied in large-scale aquaculture. *D. avara* is a softer sponge with a more fragile skeleton in comparison to bath sponges. We have cut explants and hold them on horizontal ropes, placed them in individual cages, and glued them to horizontal substrates, respectively. As a control, we also monitored growth and survival of untouched individuals in the same experimental zone. The success of the culture technique was not only evaluated by survival and growth of the sponges but also by evaluating the production of the target metabolite (bioactivity). The secondary metabolite production can vary due to both external and internal factors [29–32] and farming structures used to support sponges can affect their metabolite production [26]. Thus, survival, growth, and bioactivity of *D. avara* explants have been monitored along the year in the three above mentioned culture methods.

## 2. Material and Methods

### 2.1. Culture experimental design

The study was carried out in the western Mediterranean Sea at the locality of l’Escala, North-East of Spain (GPS coordinates: 42° 06.863’N, 003° 10.116’E) from 5 to 20 m of depth on a rocky bottom. This area was selected because of the abundance of *Dysidea avara*, what is an indicator of the good conditions of this area for the sponge culture.

A total of 75 large *Dysidea avara,* from 500 to 2,000 cm^3^ in size, living at 12–14 m of depth, were targeted as donor individuals. From them, a sponge fragment, c.a. 28 cm^3^ in size, was cut from each donor without removing the donor from its substrate. In order to minimize manipulation, the explants were submitted to the three experimental treatments (N = 25), immediately after collection, which consisted in i) to hang the sponges from ropes, ii) to place them within perforated cages or iii) to attach the sponges by glue on rigid frames (50 × 50 cm). All the treatments were placed 8 m deep in close proximity to a *D. avara* natural population.

**Figure 1:**
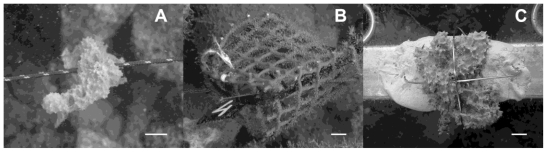
Underwater picture of the three culture methods assayed: (A) *D. avara* explant cultured by the rope method (B) cage containing a *D. avara* explant (C) *D. avara* explant glued to the metallic frame with the two tie raps holding it. The scale bar is 1 cm.

The rope method used in this study is similar to that already used by other authors [27,33], where a rope was inserted in a large needle and carefully passed through the sponge tissue. The ropes with the explants where placed horizontally, anchored to the rigid frames (Figure 1A). In the second method we placed each explant inside a 6 × 6 × 5 cm cage. The cages were made of a rigid 1 cm mesh size, plastic net to ensure seawater flow through the cages (Figure 1B). The third method consisted of gluing the explants to a horizontal steel frame with a non-toxic, two components resin (IVEGOR) (Figure 1C). Cages, frames and ropes were all placed at a distance of 40 cm from the sea bottom.

The study started in winter when the seawater low temperature favours survival during the critical period of explant attachment [28,34–36].

### 2.2. Monitoring

The cultured explants and the controls where monitored once a month for survival and at months six and ten for growth, during ten months, taking underwater pictures of each individual. Explant survival, recovery from eventual damage, and growth were estimated from the pictures. The cage cover was opened before taking pictures of the explants cultured inside cages. Bioactivity was only analysed at the end of the culture.

Survival rate (S) was calculated as the percentage of explants, which were alive at one monitoring time (N_t_) divided by the living sponge explants at the previous month (N_t + 1_):

$$\text{S}=({\text{N}}_{\text{t}+1}/{\text{N}}_{\text{t}})\times 100$$

Growth was measured as the increase in volume (V) of explants. The size of each explant was calculated by multiplying its projected area by its mean height. The mean height was computed as the average of 5 measurements (taking into account also digitations). The sponge projected area and the mean height was derived from pictures by using image analysis (NIH Image program). Growth rates at six and ten months of culture (V_6 or 10_) were calculated as the percentage of the volume at month “6” and “10” with respect to the explant initial volume (V_0_):

$${\text{GR}}_{6\;\text{or }10}=({\text{V}}_{6\;\text{or }10}/{\text{V}}_{0})\times 100$$

The error associated with the method used to calculate the explant growth prevents us from considering it as the real growth of *Dysidea avara*. However, the measurements can safely be used for comparison among the three culture techniques. The donor individuals (manipulation control) were not measured as for growth rates because of their much more complex shape with respect to that of the explants, which makes comparisons useless.

### 2.3. Toxicity analysis

To quantify the natural toxicity of the samples we used the Microtox^®^ (Microbics, Carlsbad, CA, USA), a standardised method previously described [37]. This method measures light production by the bioluminescent bacterium *Photobacterium phosphoreum*, and detects bioluminescence decreasing when the bacteria are put in contact with the crude extracts of *D. avara*. Previous studies have reported an accurate positive relationship between concentration of avarol and Microtox-measured toxicity [38].

At the end of the culture (ten months), the explants from the treatments and the control were taken to the laboratory. The samples were freeze-dried. An amount of 0.25 g of sample was squeezed in a mortar and extracted with dicloromethanol/methanol (1:1). Once the solvent was evaporated, the crude extract was weighed and resuspended through sonication in artificial seawater for the toxicity analyses by Microtox^®^. Toxicity was assayed at an initial concentration of 5 mg/mL of sponge dry weight. In every assay, a control and four decreasing concentrations (with a dilution factor of two) were tested after incubation of 5 min at 15 °C (temperature at which bacteria are active and produce bioluminescence). With these measures, a regression analysis on log/log scale between concentrations of crude extract and output of light was recorded. The EC_50_ value indicated in the regression equation is the concentration of crude extract that produces 50% in light decrease, which is assumed to represent the death of 50% of phosphorescent bacteria. The value 100/EC_50_ was calculated for each sample and used as measurement of toxicity.

### 2.4. Data analysis

Survival was analyzed using the “life tables” statistics [39]. Significant differences between size-classes were assessed by “Comparing Survival in Multiple Groups”. Then, comparison between each pair of size-classes was performed using the Gehan-Wilcoxon test. Mean growth rates after six and ten months were analysed by the non-parametric Kruskal-Wallis test because data did not comply with the normality and homoscedasticity assumptions required for parametric analyses. Differences in toxicity between specimens cultured under the three culture methods and wild specimens were analysed by One-way ANOVAs after checking the data for accomplishment of the normality and homoscedasticity assumptions (Statistica 6.0 package).

## 3. Results

### 3.1. Survival

**Figure 2:**
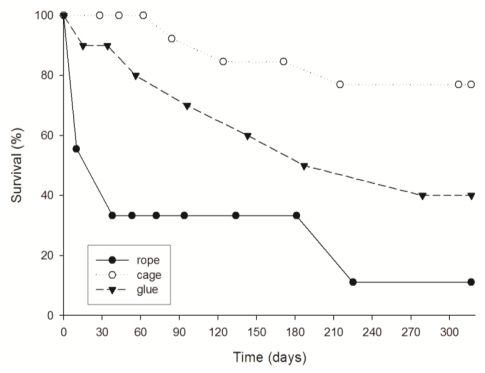
Survival (%) of *D. avara* explants cultured by the three methods.

Survival of explants was significantly different in the three culture methods used (p < 0.05, comparing survival in multiple groups (Figure 2). At the end of the culture (10 months), the explants placed in cages presented the highest survival (ca. 70% after 10 months of culture). The glued explants had an intermediate survival (ca. 40%), and the explants hold in ropes showed the lowest survival rates (11%). It is important to remark that for the explants hold in ropes the first month of culture was critical. After ten days the explants on ropes were reduced to a half, and at day 30, explant survival had already decreased to 38%.

The mortality of the explants glued to the frame and hanged from ropes was mainly due to specimen losses because of the high water turbulence. In contrast, the cage method avoided individual removing and probably enhanced protection against predators. All the specimens of *Dysidea avara* used to obtain the explants (donors) survived until the end of the monitoring (ten months) and appeared healthy.

### 3.2. Growth

**Figure 3:**
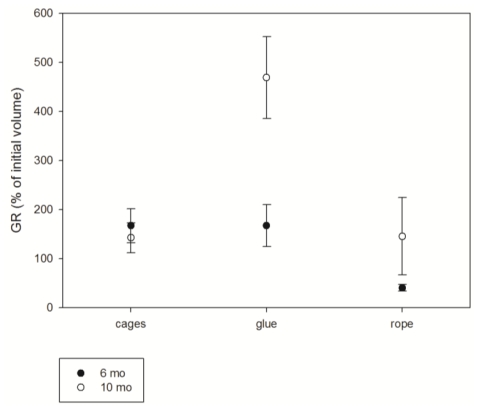
Average growth rate *of D. avara* explants cultured by the three methods at six and ten months of culture.

Although positive growth rates were registered at the end of the culture (after ten months), growth rates varied depending on the method tested (Figure 3).

**Figure 4:**
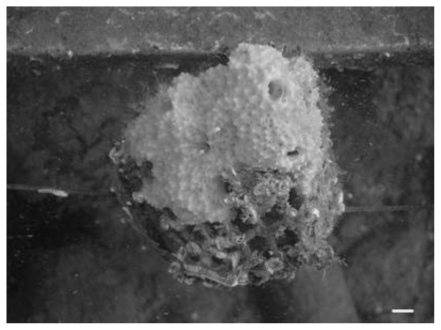
Picture of a *D. avara* explant growing out of the cage after eight months of culture. The scale bar is 1 cm.

After six months of culture, there were no significant differences among the three culture methods (p > 0.05, Kruskal-Wallis ANOVA test); however the explants cultured on ropes presented the lowest mean growth rate (ca. 40%), while the explants cultured in cages or glued to the frame presented similarly high growth rates (166.75 ± 34.62% and 167.23 ± 42.7; mean ± standard error, respectively). The explants placed in cages attached to the plastic net quite fast and after some weeks some of them grew out of the cage (Figure 4).

At the end of the culture (ten months), significant differences between the three tested culture methods were found (p < 0.05, Kruskal-Wallis ANOVA test). It is remarkable that the explants glued on the frame have increased notably in size, reaching the highest mean growth rates (468.9 ± 83.72%), while the explants cultured inside cages maintained the mean growth rate (142.34 ± 30.47%) monitored after six months of culture. On the other hand, the explants cultured on ropes, for which the lowest growth rates were recorded at month six, have grown notably after month ten (145.25 ± 79.06%), equalling the growth rate of the explants cultured in cages.

### 3.3. Toxicity

**Figure 5:**
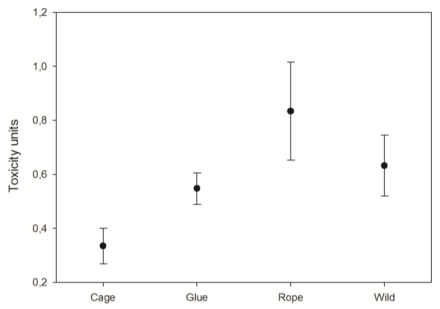
Average toxicity of the *D. avara* explants cultured by the three methods after ten months of culture and wild specimens. Vertical bars correspond to standard errors.

At the end of the experiment, the explant toxicity was significantly different in the three experimental cultures (p < 0.05, One-way ANOVA; Figure 5). The explants growing in cages presented the lowest toxicity, while the ones hanging from ropes showed the highest toxicity (p < 0.05, Fisher LSD *post hoc* test). The explants glued to the frame presented similar toxicity to the control specimens (p > 0.05, Fisher LSD *post hoc* test).

## 4. Discussion

### 4.1. Explant survival

*Dysidea avara* explants cultured in cages showed the highest survival along the whole experiment probably due to the combination of several factors: (1) the relative low manipulation of those explants; (2) the impossibility of losing explants from the cages, and (3) the physical protection against potential predators [40]. In particular, the cages avoided the high mortality due to individual losses, which occurs during the critical phase of attachment (first weeks of culture). The severity of this period depends on the species capability for attaching to the new substrate (e.g., cage, rope, and resin) and can be enhanced by a high level of sponge manipulation [33]. Conversely, the sponges cultured on ropes presented a high mortality (ca. 70%) during the first 30 days because they show a particular difficulty to attach to the rope. On the other hand, the glued explants experienced an intermediate level of manipulation (less than the rope cultured explants and more than the ones placed in cages) what probably explains their middle mortality rates.

We observed that the high water flow present in our study area, although suitable for the sponge growth, becomes a critical factor for survival of explants on ropes due to substrate instability and the associated reduction in attachment success. In our study area (8 m of depth) the water flow ranges from 0.05 to 0.15 m/s, and occasionally peacks to 0.66 m/s [41]. These water flow conditions are comparable to the ones described by Duckworth and Battershill in an exposed site (from 0.19 to 46 m/s) where two sponge species (*Polymastia croceus* and *Latrunculia wellingtonensis*) where cultured by several different methods at 12 m deep [27]. Duckworth and Battershill (2003) also found that strong water movement tore some explants off the rope, leading to survival rates of 59% and 22%, after nine months of culture for *P. croceus* and *L. wellingtonensis*, respectively. Moreover, explants cultured in cages at the end of the experiment (ten months) had survival rates (ca. 77%) comparable to those after nine months of *P. croceus* and *L. wellingtonensis*, cultured in mesh (96% and 61%, respectively) [27].

Survival rates depend on the species features. It has been speculated that the spongine contents of the sponge tissue plays an important role in the capability of regeneration and potential attachment of the target sponge; conversely the sponges with spicule skeletons and low collagen content have less capability to recover from manipulation and have more difficulties for attaching to the new substrate [39]. *Dysidea avara* is a relatively elastic sponge but has not as much spongine as bath sponges (e.g., *Spongia officinalis*), what makes alternative culture methods as cages or glue, more suitable (higher survival) than ropes for the farmers.

### 4.2. Explant growth

As for explant growth, gluing the explants to horizontal solid substrates such a metallic frame seemed to be the best method to culture *Dysidea avara* in the sea. At the end of the experiment (ten months) the explants glued to the substrate had the largest final size. This huge growth rate, which can be considered high when compared with that of other cultured sponges (Table 3 in [27]), has to be taken with care because of the method used for growth measuring. The high growth recorded may be a consequence of the non-invasive nature of the gluing method and the lower stress it produced to the sponges, compared with cages and ropes. Moreover, the glue provides a new artificial substrate, which seems to be suitable for a faster and easy attachment of the explants.

The caged sponges, despite the low manipulation they suffered, showed similarly low growth rates to the ones cultured by ropes at the end of the experiment. This low growth rate can be attributed to several causes. The initial high growth rate could be favoured by the presence of the new substrate (*i.e.*, the rigid net); however after the explant tissue engulfed the net, no longer substrate was available to extend on. On the other hand, fouling organisms, settled after some months of culture on the net-made cages, reduced water flow through the caged sponge. This flow reduction may represent a trophic depletion for the sponge, which may have hampered growth.

Some generalization can be envisaged as for *Dysidea avara* growth: high growth rates have been recorded with the three methods assayed, but high growth variability has also been recorded among explants cultured under the same method. Thus intraspecific growth variability seems to be a common feature to both sponges under culture and wild specimens [35,42,43]

The relatively high growth rates obtained for the cultured sponges compared with the previous reported values for other species [18,44] are probably due firstly to the particular species dynamism but secondly to the favourable hydrodynamics of the zone (e.g., strong water flow and high concentration of food particles in the water). Other authors have already stated that water flow intensity can greatly affect the growth of cultured sponges [27]. In particular, high water movement generally promotes high growth through increased food availability [25,27,34].

### 4.3. Explants toxicity

The lowest bioactivity presented by the explants cultured in cages is coincident with the low growth rates shown by these explants, which might be the result of a lower water flow across the cages and a consequent reduction of the available food for the explants. In contrast, the highest bioactivity was shown by explants handling from ropes, which is maybe due to the higher stress that this unstable substrate produced on the sponges.

The explants glued to the substrate presented a similar bioactivity to the wild specimens, which points to this method as the best culture method for *Dysidea avara* culture, when obtaining secondary metabolites is attempted. However, investigation on the exact environmental factors that enhance metabolite production should be addressed before a culture method can be seriously proposed.

## 5. Conclusions

When we consider survival, growth, and bioactivity of the cultured sponges altogether, it is made evident that the three variables are not positively correlated. While cages represent the best method for explant survival, gluing the explants is the best method for obtaining the highest growth and handling the explants from ropes produced the highest bioactivity. In our case, with all the results at hand, we will recommend to culture *Dysidea avara* explants with the glue method. Despite glued explants presented a higher mortality than the ones cultured in cages, their growth rates and their bioactivity compensated those losses. Moreover, for this species and under the environmental conditions assayed, we can propose the sponge aquaculture as a suitable alternative to harvesting those sponges from natural populations.

The results of our assays indicate that it is highly important to select the most appropriate method, before starting a sponge culture. The best method will depend on the sponge species and the environmental characteristics of the culture location. This implies a previous knowledge of the biology and physiology (e.g., elasticity, recovery capability, growth) of the target species and the environmental conditions of the selected zone (e.g., water flow, T, *etc.*). A consensus between survival and growth must be achieved because generally the conditions that increase sponge growth (e.g., high water flow) are also the ones that increase explant mortality. Moreover, increasing the production of secondary metabolites of the sponges under culture has also to be considered. Finally, sponge growth rates were very different at six months that at ten months, which highlights the necessity of long-term experimental cultures for providing an accurate overview of the culture development. This especially applies when the target species are sponges with important variations in growth rates and mortality with time.
